# Use-dependent increase in attention to the prosthetic foot in patients with lower limb amputation

**DOI:** 10.1038/s41598-022-16732-z

**Published:** 2022-07-23

**Authors:** Naoki Aizu, Yutaka Oouchida, Kouji Yamada, Kazuhiro Nishii, Shin-Ichi Izumi

**Affiliations:** 1 Department of Physical Medicine and Rehabilitation, Tohoku University, Miyagi, Japan; 2 Faculty of Rehabilitation, School of Health Sciences, Fujita Health University, Aichi, Japan; 3 Department of Education, Course for Special Needs Education Teachers, Osaka Kyoiku University, Osaka, Japan; 4 Graduate School of Biomedical Engineering, Tohoku University, Miyagi, Japan

**Keywords:** Neuroscience, Neurology

## Abstract

Patients with lower limb amputation experience “embodiment” while using a prosthesis, perceiving it as part of their body. Humans control their biological body parts and receive appropriate information by directing attention toward them, which is called body-specific attention. This study investigated whether patients with lower limb amputation similarly direct attention to prosthetic limbs. The participants were 11 patients with lower limb amputation who started training to walk with a prosthesis. Attention to the prosthetic foot was measured longitudinally by a visual detection task. In the initial stage of walking rehabilitation, the index of attention to the prosthetic foot was lower than that to the healthy foot. In the final stage, however, there was no significant difference between the two indexes of attention. Correlation analysis revealed that the longer the duration of prosthetic foot use, the greater the attention directed toward it. These findings indicate that using a prosthesis focuses attention akin to that of an individual’s biological limb. Moreover, they expressed that the prosthesis felt like a part of their body when they could walk independently. These findings suggest that the use of prostheses causes integration of visual information and movement about the prosthesis, resulting in its subjective embodiment.

## Introduction

Patients with lower limb amputation re-learn walking with a prosthetic foot during rehabilitation to compensate for the lost limb. As they become more proficient in using prostheses, they are even able to run and climb mountains with them. When learning to use a prosthesis, many patients experience “embodiment”, which refers to the feeling that the prosthesis is a part of their own body. Recent studies reported that increased use of a prosthesis led to its increased perception as part of the body^1,2^. Therefore, embodiment of the prosthesis is important for walking rehabilitation^3,4^. However, the process of embodiment in patients with lower limb amputation is unclear. Patients with amputation who can walk with a prosthesis have higher survival rates and lower rates of re-amputation than do those who cannot walk, making it important for these patients to understand how they should use their prostheses during walking rehabilitation^5,6^.

To use the prosthetic foot well, patients with lower limb amputation may direct their attention to it, as they would to their own body part. Attention is a neural mechanism for the selection of behaviorally relevant information from a multitude of perceptions^7–9^. To perform the intended movement accurately, the brain directs attention to the natural body parts. Previous reports have shown that the brain updates information about the body by monitoring information such as its position^10,11^. Our previous study also reported that a visual detection task can quantitatively measure body-specific attention^12^ using the body facilitation effect. This effect can be explained by the fact that a target present on the body is detected faster compared to one situated far from the body^13–18^. This phenomenon indicates that the brain directs attention to one’s own body and regulates the amount of information from the limb to aid in motor control, and attention is potentially directed to the natural body parts^16^. Moreover, this body-specific attention is top-down in nature, i.e., it is an intrinsic process that that spontaneously directs attention to body parts in a space^12,14–16^. Interestingly, after healthy adults have been using a prosthetic hand well, this body facilitation effect was also found to act on the prosthesis. However, this effect was not observed before they used the prosthesis^13,14^. Moreover, animal studies showed that body representations in the brain can be modified when using a tool, which is then incorporated and becomes a part of the organism’s body^19,20^. These findings seem to indicate that patients with lower limb amputation who use their prosthesis well, direct more attention to the prosthetic foot, as they experience the prosthetic foot as their own body part; however, evidence regarding this is unclear.

This study aimed to examine whether attention is directed toward the prosthetic foot. We hypothesized that if patients with amputation could use their prosthetic limbs well for walking, the attention directed toward the prosthesis would increase. Furthermore, the principal factors and clinical features that could explain the changes in the attention directed toward the prosthesis were identified.

## Results

**Figure 1:**
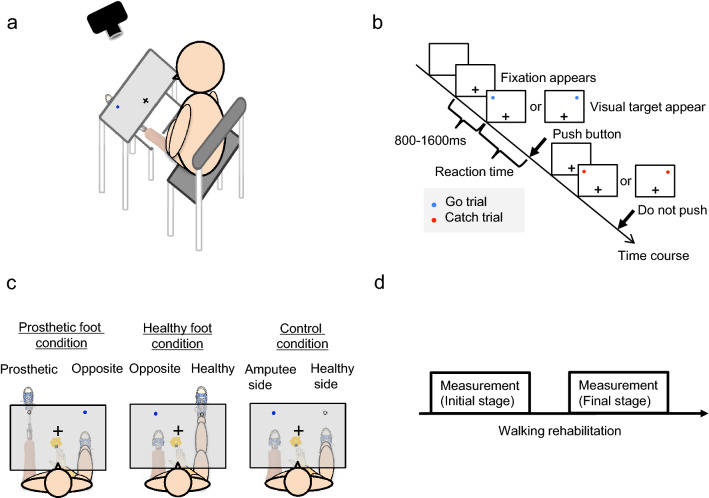
Experimental design. The figure demonstrates the experimental task and procedure in four panels. (**a**) Experimental environment: Patients sat in a chair in front of a rack in a quiet room and were required to respond to a visual target that appeared on either the prosthetic foot or on the opposite side from a projector attached to the rack. (**b**) Visual detection task: After the fixation point appeared, the visual target randomly appeared from 800 to 1600 ms on a white board. Patients were required to push the button only when a blue-filled circle appeared. (**c**) Experimental conditions: In the prosthetic foot condition, patients placed their prosthetic foot on a foot-stand. In the healthy foot condition, their healthy foot was placed on a foot-stand. In the control condition, neither the prosthetic foot nor the healthy foot were placed on a foot-stand. For patients with left lower limb amputation, in the prosthetic foot condition, the left prosthetic foot was placed on a foot-stand on the left side. (**d**) Procedure: The measurements were performed in two periods. The initial stage refers to the time at which one month had passed since patients began using the prosthetic foot and they were able to walk with the supervision of medical staff. The final stage is the time when they could walk independently.

To test attention to the prosthetic foot at the initial and final stages of rehabilitation among patients with lower limb amputation (Table 1), participants engaged in a visual detection task (Fig. 1, further details are given under Methods), from which the “index of attention to prosthetic foot” was calculated (see Analysis under Methods).

**Table 1:** Clinical characteristics of the patients.

No	Age	Sex	Amputation side	Amputation site	Cause	Index of attention to prosthetic foot (msec)		Walking speed (m/s)		Assistive devices (number)	
						Initial phase	Final phase	Initial phase	Final phase	Initial phase	Final phase
1	50	M	Right	A/K	Tumor	− 23.5	11.8	1.04	1.24	2	1
2	62	F	Right	A/K	Accident	− 16.0	− 6.0	0.61	1.23	1	0
3	72	M	Left	A/K	Accident	− 36.6	86.0	0.62	0.74	1	1
4	61	F	Right	B/K	PAD	10.4	34.0	0.79	0.78	1	1
5	18	M	Left	B/K	PAD	− 58.6	− 35.1	1.05	2.33	0	0
6	51	M	Left	B/K	PAD	2.7	13.2	0.63	0.77	1	1
7	74	F	Left	B/K	PAD	− 39.4	− 35.5	0.29	1.15	1	0
8	83	M	Left	A/K	Tumor	− 67.9	13.5	0.38	0.35	1	1
9	43	M	Left	A/K	Tumor	− 25.7	− 5.4	1.04	1.47	2	1
10	36	F	Left	HD	Tumor	− 51.1	− 23.7	0.48	0.53	2	1
11	36	M	Left	A/K	Tumor	− 34.3	− 31.0	1.09	1.61	2	0

*M* Male, *F* Female, *A/K* Above-knee amputation, *B/K* Below-knee amputation, *HD* Hip disarticulation amputation, *PAD* Peripheral vascular disease.

**Figure 2:**
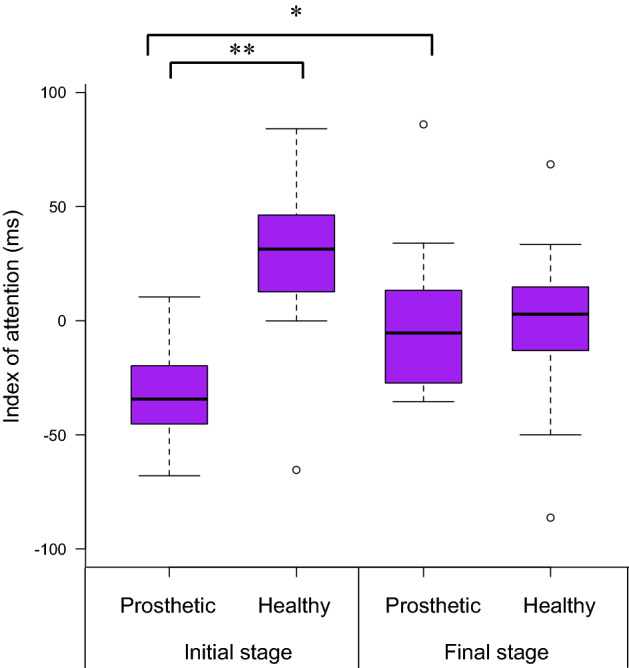
Change in index of attention to the prosthetic foot and healthy foot. The box plot shows the attention data for each foot and phase. In the initial stage, the index of attention to the prosthetic foot was lower than that to the healthy foot, while in the final stage, there was no significant difference between the index of attention to the prosthetic foot and that to the healthy foot. The index of attention to the prosthetic foot in the final stage was higher than that in the initial stage. There was no significant difference in the index of attention to the healthy foot between the initial and final stages. The circle indicates an outlier. * *p* < 0.05, ** *p* < 0.01.

To clarify the change in the index of attention to the prosthetic and the healthy foot, as a result of improved walking ability, we performed a two-way analysis of variance (ANOVA) with repeated measures. Results from the ANOVA found a statistically significant interaction between the stage and foot factors (F_1,10_ = 6.190, *p* = 0.032) and no main effect for both factors (stage: F_1,10_ = 0.034, *p* = 0.858, foot: F_1,10_ = 4.032, *p* = 0.072). In multiple comparisons with the Bonferroni correction, the index of attention to the prosthetic foot (mean: − 30.92 ms) was lower than that to the healthy foot (mean: 27.80 ms) in the initial stage (*p* = 0.005; Fig. 2). Additionally, the index of attention to the prosthetic foot in the final stage was higher than that in the initial stage (*p* = 0.014), and there was no significant difference between the indexes of attention to the prosthetic foot (mean: 1.98 ms) and healthy foot (mean: − 2.21 ms) in the final stage. Conversely, the average number of incorrect responses to a red-filled circle, which required no response, was 0.83 and 0.69 times in the initial and final stages, respectively. There was no statistical difference in the number of incorrect responses between the initial and final stages. G*Power (Version 3.1.9.6)^21^ was used to analyze the sample size based on the interaction of the index of attention to the prosthetic foot and the healthy one. The results showed that the number of patients who participated in this study sufficiently met the recommended sample size.

**Figure 3:**
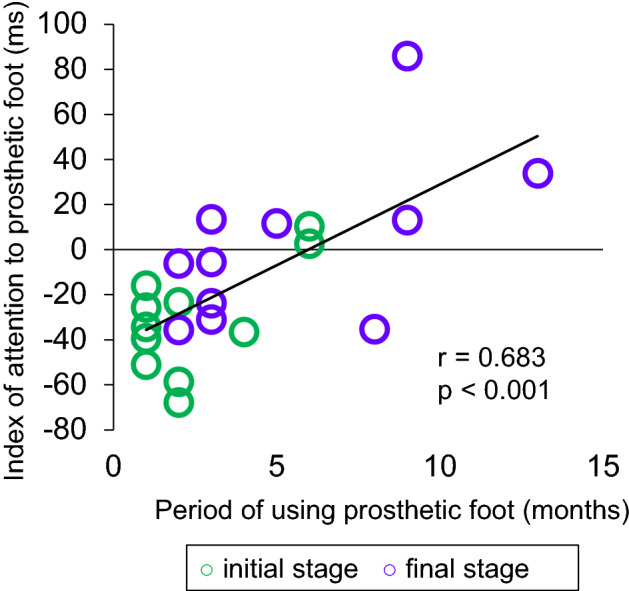
Correlation analysis of the index of attention to the prosthetic foot: initial and final stages. This figure clarifies the relationship between the index of attention and the period of using the prosthetic in the initial and final stages. The index of attention to the prosthetic foot was positively correlated with the period of using the prosthetic foot (r = 0.683, *p* < 0.001). Individual patients are represented by circles, with the green circle representing the initial stage (*N* = 11) and the purple one the final stage of rehabilitation (*N* = 11).

To clarify the relationship between the index of attention to the prosthesis and other characteristic data, a correlation analysis was performed with the initial and final stage data. The analysis showed statistically significant correlations between the index of attention to the prosthetic foot and the period of using the prosthetic foot (r = 0.683, *p* < 0.001, Fig. 3).

**Figure 4:**
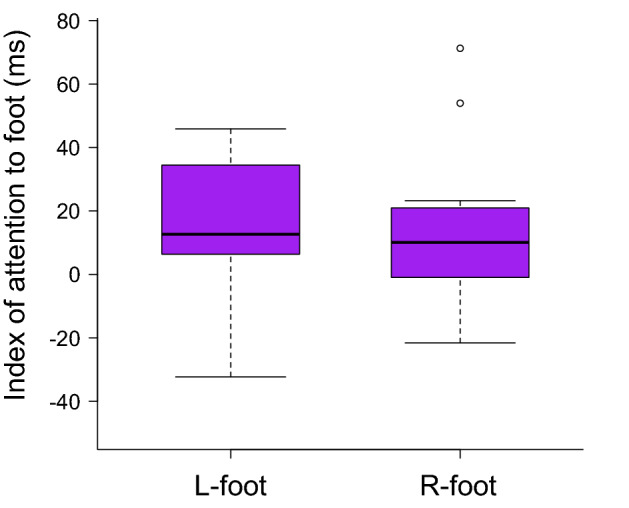
Box plots showing index of attention to the left and right foot in healthy adults. There was no significant difference between the index of attention to the left foot (L-foot) and right foot (R-foot). Circles represent outliers.

To confirm the facilitation of visual stimulus detection on the foot among healthy adults, their body-specific attention between the left and right foot was measured using a visual detection task. The results showed an enhancement in visual stimulus detection for both the left and right foot. Moreover, the index of attention to the left and right foot was calculated (mean: left foot = 12.03 ms, right foot = 9.43 ms). No difference was found between the index of attention to the left and right foot (paired *t*-test, *t* = − 0.047, *p* = 0.964, Fig. 4).

**Figure 5:**
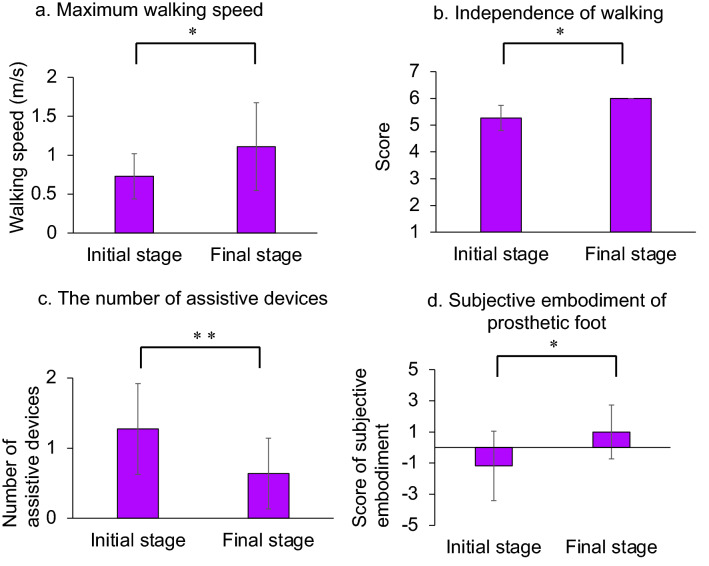
Comparison of initial and final stages. Figure clarifying the change in walking speed, independence in walking, number of assistive devices, and subjective embodiment of the prosthetic foot from the initial stage to the final stage. From the initial phase to the final phase, walking speed increased (**a**), walking independence score increased (**b**), the number of assistive devices decreased (**c**), and the score of subjective embodiment increased (**d**). Error bars represent standard deviations. * *p* < 0.05, ** *p* < 0.01.

To clarify the change in walking ability with the prosthetic foot from the initial stage to the final stage, the maximum walking speed and number of assistive devices in walking as well as independence of walking was assessed using the Functional Independence Measure (FIM)^22,23^ at both stages. Results showed an increase in maximum walking speed (paired t-test, *p* = 0.012, mean: initial stage 0.73 m/sec, final stage 1.11 m/sec, Fig. 5a) along with an increase in the FIM score for walking (Wilcoxon, *p* <  = 0.005, median: initial stage 5, final stage 6, Fig. 5b) and a decrease in the number of assistive devices used (Wilcoxon signed-rank test, *p* = 0.020, median: initial stage 1, final stage 1, Fig. 5c). Therefore, the results demonstrated an improvement in the walking ability from the initial to the final stages.

To assess subjective change from the initial to the final stage, patients answered questionnaires on the subjective embodiment of the prosthetic foot and phantom limb vividness and pain during the two periods. From the initial to the final stage, the subjective embodiment score of the prosthetic foot increased (Wilcoxon, *p* = 0.011, median: initial stage − 1, final stage 1, Fig. 5d), implying that patients strongly felt that the prosthetic foot was their own foot in the final stage. Meanwhile, there was no statistically significant difference in the vividness experience of the phantom limb and the severity of phantom limb pain between the initial and final stages.

## Discussion

To clarify the direction of attention in patients with lower limb amputation, the present study longitudinally evaluated the attention directed to the prosthetic foot using a visual detection task among 11 participants. Walking ability, maximum walking speed, number of assistive devices used in walking, and independence of walking in FIM improved in the final stage when patients could walk independently, compared to the initial stage when they were only able to walk with supervision. Additionally, the index of attention to the prosthetic foot was lower than that to the healthy foot in the initial stage, while the index of attention to the prosthetic foot increased in the final stage compared with the initial stage. Moreover, there was no significant difference between the index of attention to the prosthetic foot and the healthy foot in the final stage. Our correlation analysis showed that the index of attention to the prosthetic foot correlated with the period of using the prosthetic foot, indicating that the longer the duration of prosthetic foot use, the more attention patients directed toward it. These findings indicate an adaptive change in the patient’s direct attention to the prosthetic foot in a use-dependent manner. Moreover, the subjective embodiment score of the prosthetic foot in the final stage was higher than that in the initial stage. These findings suggested that the use of prostheses causes the integration of visual information and movement about the prosthesis, resulting in subjective embodiment.

The results found that attention to the prosthetic foot was low in the initial stage; however, this attention increased in the final stage. Moreover, the index of attention to the prosthetic foot correlated with the duration of prosthetic foot use, indicating that patients gradually directed attention to the prosthetic foot when using prostheses. A previous report showed that when any of the sensory inputs (for example, the visual, somatosensory, and vestibular inputs) are absent or inaccurate, the central nervous system adjusts the gains for each input to control stability^24^. When visual information is blocked, patients with limb amputation have significantly less motor control than do healthy individuals^25–28^. A recent study identified stronger functional connectivity between visual and sensorimotor areas in individuals who used their prostheses more frequently, suggesting that altered daily motor behavior facilitates prosthesis-related visual processing^29^. These findings indicate that patients with limb amputation rely heavily on visual information related to their prostheses to use the tool more effectively. Additionally, the use of a visual detection task in psychophysics studies reveals that the experience of functional tool-use facilitates visual information processing related to the tool in healthy adults^13,14^. Therefore, these findings suggest a strategy to promote visual information processing in which patients with lower limb amputation direct their attention to the prosthetic foot for useful visual information from the prosthesis, thus resulting in its better use.

Further, the results showed that the subjective embodiment score of the prosthetic foot in the final stage was higher than that in the initial stage, indicating that patients felt that the prosthetic foot was their own body part. Previous studies have reported that the subjective embodiment of a prosthesis, such as a rubber hand, is generated by multi-sensory integration, including not only visual and somatosensory input^30^ but also active movement^31,32^. Given the limited somatosensory information from the prosthesis, it is suggested that use of prostheses causes the integration of visual information and movement of the limb, including the prosthetic foot, resulting in its subjective embodiment. Recent reports have shown that increasing sensory information can be beneficial for walking, as it lightens the perceived prosthesis weight^33^, increases walking speed, and decreases mental and physical fatigue during walking^34^. Moreover, our results showed that there was no significant difference in the index of attention between the prosthetic foot and the healthy foot in the final stage. These findings suggest that using the prosthesis causes attention to be directed to it, to a degree like that of one’s own body, and that the embodiment of the prosthesis does occur.

Although the results showed that the subjective embodiment of the prosthesis increased in the final stage—compared with the initial stage—several patients struggled to answer the item “I felt as if the prosthetic foot was my foot.” Several participants made the following remarks: “I cannot live positively unless I understand that I lost my foot” and “I can use the prosthetic foot successfully, but the prosthetic foot cannot move like my previous foot.” In a previous study, patients expressed that a prosthesis is a tool that does not belong to the body, while others stated that the device becomes an integral part of their physical selves, suggesting that prosthesis users differ in their embodiment experience of prosthesis use^35^. In our results, the index of attention to the prosthetic foot did not correlate with the subjective embodiment score, suggesting that there is a discrepancy between implicit and explicit cognitive processes. A previous study showed that patients with motor paralysis decreased their body-specific attention to the paretic hand, suggesting that they used the intact limb instead of the paretic limb to perform an action by actively inhibiting the use of the paretic limb, resulting in decreased attention to or neglect of the paretic limb^12^. The present results also showed that there was no significant difference in the index of attention between the prosthetic foot and healthy foot in the final stage, indicating that patient’s direct attention to the prosthetic foot as well as to the healthy foot. These findings suggest that attention to the prosthetic foot is a specific index that can objectively measure the embodiment of the prosthesis.

Longitudinal measurement of attention to the prosthesis in patients with lower limb amputation may lead to shorter hospitalization and rehabilitation periods; this is an important consideration for future research and practice. Results from this study showed that attention to the prosthesis increased at the time of hospital discharge or at the end of rehabilitation. This implies that attention to the prosthesis is helpful in controlling gait while using the prosthesis. It also demonstrates the patient’s proficient walking ability. It is important to note that in the initial stage, patients managed to walk with many assistive devices but did not direct attention to their prosthesis, as if they were ignoring it. In the initial stage, it is difficult even for rehabilitation therapists to determine whether the patient is walking independently, due to their increased risk of sudden falling. Therefore, clarifying the amount of attention to the prosthesis may be an important factor in objectively predicting the proficiency of walking ability. Objective judgments of gait proficiency can be used as a guideline for the duration of hospitalization and rehabilitation, which is also beneficial to the medical economy. Thus, the amount of attention directed to the prosthesis is one indicator for further research that seeks to determine which interventions of rehabilitation or which prosthetic parts and their respective functions will better facilitate the acquisition of prosthetic gait.

Previous studies have reported that motor performance and motor learning processes differ, depending on whether explicit attention is directed to the body or to the environment^36,37^. Many studies have suggested that explicit attention should be directed to the external environment. However, some studies have also reported individual differences, suggesting that the optimal location of attention may differ, depending on the participant^38–40^. However, this study reveals that it is not clear whether explicit attention should be directed to the body or to the environment when attention to own body parts or prostheses is reduced. These findings provide novel information for the study of explicit attention, which is useful for motor control.

From our results, it is unclear whether the amount of attention to the prosthesis is appropriate, and it is recommended that its relationship with other indicators, such as the frequency of falls, is considered. In the future, clarifying the body-specific attention of prosthetic legs of proficient users, that is, patients who use a prosthesis for a long period of time and sports athletes, will help to understand the amount of appropriate attention necessary for patients with limb amputation. Furthermore, the measurement of prosthetic foot attention in proficient users of prostheses may provide important insights into the mechanisms of prosthetic foot control.

There were several limitations to this study. First, the results showed that the duration of prosthesis use was not correlated with walking speed. Walking speed was the only walking-related parameter assessed in this study, in which the stability indices were not measured. Our results demonstrated a decrease in the frequency of cane use and an increase in walking independence from the initial phase to the final phase; thus, we can infer that the stability of walking with the prothesis increased gradually. Furthermore, a recent study^16^ reported a relationship between toe grasp strength, which contributes to stability^41–43^, and body-specific attention in the foot in healthy adults. Future studies should conduct in-depth investigations into the walking parameters that are related to body-specific attention toward the prosthetic foot in patients with amputation. Second, since there was bias due to the amputation side and amputation site in the participations, it was difficult to discuss the differences between them. A larger sample size is required for future investigations, because the side of the amputation affects body representation in the brain^44^, and the amputation site directly affects walking ability.

This study showed that use-dependent increased attention to the prosthetic foot was found in patients with lower limb amputation. This is the first study to reveal the strong relationship between attention to the prosthetic foot and walking ability with the prosthesis by using psychophysiological techniques. This study also succeeded in showing an adaptive change in attention to the lower limb, which explains the embodiment mechanism of the prosthesis. Increased attention to the prosthetic foot is an important factor of walking rehabilitation. Further studies should discover ways to increase attention to the prosthetic foot to facilitate walking ability in patients with lower limb amputation.

## Methods

### Participants

This longitudinal study was performed with data that were prospectively collected from Tohoku University Hospital, Japan from 2012 through 2017. Fourteen patients who underwent amputation were initially recruited. From these, three patients who had serious uncontrolled medical conditions (e.g., cancer recurrence) during the experimental period and for whom data could not be obtained in the final phase were excluded. Finally, 11 inpatients and outpatients with lower limb amputation participated in this study [mean (± SD) age = 53.2 (± 19.3) years; seven men, all of whom were right-handed; Table 1]. Participants received physical therapy at Tohoku University Hospital, Japan to re-learn walking using a prosthetic foot. The levels of amputation were transtibial (below-knee, n = 4) and transfemoral (above-knee, n = 6), in addition to one hip disarticulation; the causes of amputation were tumor, peripheral vascular disease, and trauma in five, four, and two patients, respectively. The patients used walking devices (axillary crutch, forearm crutch, or T-cane), depending on their walking ability, during the experimental period. We also recruited 11 age-matched healthy adults as a control group (mean [± SD] age = 41.1 [± 16.8] years, seven males, 10 right-handed). The Tohoku University ethics committee approved this study (ID 2011-572, 2014-1-728). This study was conducted according to the ethical standards of the Declaration of Helsinki. Prior to the experiment, all participants agreed to participate and provided written informed consent.

### Procedure

The measurements were administered in two periods (Fig. 1d). The initial stage was the time in which one month had passed since patients began using the prosthetic foot in rehabilitation and they managed to walk with the supervision of medical staff. The final stage was the time during which it became possible for patients to walk independently without supervision. This stage was at the end of rehabilitation, immediately before getting discharged from the hospital. They used a prosthetic foot almost every day from the initial to the final stage to re-learn walking. In the initial and final stages of walking rehabilitation, attention to the prosthetic foot and healthy foot was assessed using a visual detection task. Moreover, subjective embodiment of the prosthetic foot was assessed using the item “I felt as if the prosthetic foot was my foot.” Furthermore, assessments of the walking ability, maximum walking speed, independence of walking in FIM^22,23^, number of assistive devices used, and patient characteristics—such as vividness and pain of phantom limb—were made in two stages.

### Attention to the prosthetic foot

To measure attention to the prosthetic foot in patients, a visual detection task was used, which was designed for measuring body-specific attention to detect a visual target near the body and has previously been tested on healthy adults^12–18^. Patients sat in a chair in front of a rack in a quiet room and were instructed to respond as quickly as possible to a visual target that appeared on either the prosthetic foot or on the opposite side (Fig. 1a, c). Reaction time was defined as the time between the onset of the visual target and the reaction of the participants pushing the button (Fig. 1b).

In the visual detection task, a white board covered the patients’ legs to equalize the visual information. We projected a visual target and a fixation point on the white board with a projector attached to the rack. The response button was located at the same distance from each visual target on the white board and at the center of the patient. The participants pushed a button with the right index finger as soon as the visual target appeared. A blue-filled circle, representing a “go” visual target, appeared in 80% of the trials. In the rest of the trials, a red-filled circle representing a “no-go” target that required no response appeared. The visual target had a visual angle of 1.7°, and was located 21 cm from the midsagittal plane. The distance between the fixation and the projected visual target was 32 cm, and the distance from the projected visual target to the participants was approximately 65 cm, depending on the length of their lower extremities. At the beginning of each trial, the patients were instructed to gaze at the fixation point (Fig. 1b). A visual target appeared randomly from 800 to 1600 ms after the fixation point appeared on the table, and the position of the visual target was either left or right at random. In the experiment, the patients performed 80 trials for each condition (prosthetic foot condition, healthy foot condition, and control condition). Before the experiment, the patients performed 60 trials as a training for the visual detection task. The order of each condition was counterbalanced across the participants.

### Condition

To measure attention to the prosthetic foot, the conditions were defined as prosthetic foot, healthy foot, and control conditions (Fig. 1c). In the prosthetic foot condition, the patient placed their prosthesis on the foot-stand (knee extension), with the prosthetic foot positioned such that the visual stimulus was located at the center of the prosthesis ankle. The healthy foot was placed on the ground. In the healthy foot condition, the patient placed the healthy foot on the foot-stand (knee extension), with the healthy foot positioned where the visual stimulus on the healthy side was presented. In the control condition, the prosthetic foot and healthy foot were both on the ground, and participants responded to visual stimuli to confirm the attentional bias between the amputated and healthy sides. To be precise, when the patient with the left lower limb performed the visual detection task in the prosthetic foot condition, the left prosthesis was positioned below the left visual stimulus.

### Subjective embodiment of the prosthetic foot

Subjective embodiment of the prosthetic foot was assessed by asking the following question in Japanese: “I felt as if the prosthetic foot is my foot.” Patients rated the subjective embodiment score of the prosthetic foot on an 11-point Likert scale ranging from − 5 (I completely disagree) to + 5 (I completely agree). This method of assessing subjective embodiment has been described in a previous study^45^.

### Maximum walking speed

Maximum walking speed was measured by asking participants to walk 14 m and recording the time it took for them to walk 10 m (excluding 2 m at the start and at the end). The participants were instructed to walk as quickly as possible. The time data were averaged and calculated as walking speed (m/s).

### FIM

Independence of walking was measured using a sub-item of FIM^22,23^. The FIM scores range from 1 to 7 (7: complete independence, 6: modified independence [using device], 5: supervision [without assistance], 4: minimal assistance, 3: moderate assistance, 2: maximal assistance, 1: total assistance or not testable). Scores below six required another person for supervision or assistance.

### Number of assistive devices

The patients used assistive devices (axillary crutch, forearm crutch, or T-cane) during walking to support the body. The number of assistive devices used was counted in the initial and final stages; the fewer assistive devices used, the better the ability to walk with a prosthesis.

### Vividness of the phantom limb and intensity of phantom limb pain

Patient characteristics, such as the vividness of the phantom limb and the intensity of phantom limb pain, were assessed using a questionnaire. Patients rated the vividness of the phantom limb and intensity of the phantom limb pain using an 11-point numerical rating scale from 0 (no) to 10 (worst imaginable pain or very vivid phantom limb). These methods of assessing the vividness of the phantom limb and the intensity of phantom limb pain were reported in a previous study^46^.

### Analyses

**Figure 6:**
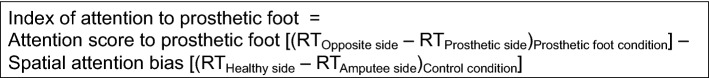
Formula for calculating the index of attention to the prosthetic foot. RT: reaction time.

The index of attention to the prosthetic foot was calculated by excluding the spatial attention bias specific to amputation^47^ (refer to Fig. 6). First, the attention score was calculated by subtracting the reaction time for the target in the prosthetic foot position from that of the opposite side. Second, spatial attentional bias was subtracted from the reaction time on the healthy side and the amputation side in the control condition. We then subtracted the spatial attentional bias from the attention score, based on previous reports that patients neglected the space near their missing limb^47^. Similarly, the index of attention to the healthy foot was also calculated.

To elucidate the attention to the prosthetic foot, we performed a two-way ANOVA with repeated measures (foot and stage), and multiple comparisons as a post hoc test with Bonferroni correction. To test the relationship between the index of attention to the prosthetic foot and the healthy foot, we performed a correlation analysis with both the initial and final stage data.

Furthermore, to examine which amputation-specific factors affected the index of attention to the prosthetic foot, we analyzed the correlation between the index of attention to the prosthetic foot and clinical characteristics of patients, such as age, duration of using the prosthetic foot, subjective embodiment of the prosthetic foot, maximum walking speed, vividness of the phantom limb, and phantom limb pain, with Pearson product-moment correlation coefficients or Spearman’s rank correlation coefficients. Additionally, to clarify the change from the initial stage to the final stage, we analyzed the maximum walking speed, subjective embodiment of the prosthetic foot, vividness of the phantom limb, and phantom limb pain in the initial and final stages using paired t-test or Wilcoxon signed-rank test. All data were checked for normality by Shapiro–Wilk test and then statistical analyses were performed. Statistical significance was set at *p* < 0.05.
