# Author Correction: Use-dependent increase in attention to the prosthetic foot in patients with lower limb amputation

**DOI:** 10.1038/s41598-022-17654-6

**Published:** 2022-08-02

**Authors:** Naoki Aizu, Yutaka Oouchida, Kouji Yamada, Kazuhiro Nishii, Shin-Ichi Izumi

**Affiliations:** 1 Department of Physical Medicine and Rehabilitation, Tohoku University, Miyagi, Japan; 2 Faculty of Rehabilitation, School of Health Sciences, Fujita Health University, Aichi, Japan; 3 Department of Education, Course for Special Needs Education Teachers, Osaka Kyoiku University, Osaka, Japan; 4 Graduate School of Biomedical Engineering, Tohoku University, Miyagi, Japan

Correction to: *Scientific reports* 10.1038/s41598-022-16732-z, published online 23 July 2022

The original version of this Article contained an error in the spelling of the author Shin-Ichi Izumi which was incorrectly given as Izumi Shin-Ichi.

The original Article has been corrected.

